# When Work Overload Cuts Both Ways: Job Crafting, Emotional Exhaustion, and the Role of Self-Efficacy in Rural Teachers’ Subjective Well-Being

**DOI:** 10.3390/bs16091684

**Published:** 2026-09-18

**Authors:** Zicheng Wang, Sizhen Chen, Huiting Liu, Tianfeng Li

**Affiliations:** 1 School of Education, Hunan University of Science and Technology, Xiangtan 411201, China; zicw@hnust.edu.cn (Z.W.); 18570550376@163.com (S.C.); 2 School of Public Administration, Huazhong University of Science and Technology, Wuhan 430074, China; d202381662@hust.edu.cn; 3 School of Humanities, Central South University, Changsha 410083, China

**Keywords:** work overload, subjective well-being, rural teachers, job crafting, emotional exhaustion, self-efficacy

## Abstract

Rural teachers often face work overload arising from demanding teaching responsibilities and extensive non-teaching duties, which may be closely associated with their subjective well-being. However, prior research has largely adopted a single-effect perspective, paying insufficient attention to the potentially competing mechanisms linking work overload to well-being. Drawing on cognitive appraisal theory of stress, this study develops a moderated parallel mediation model to examine the dual pathways through which work overload is associated with rural teachers’ subjective well-being. Survey data were collected from 639 rural primary and secondary school teachers across 12 provincial-level regions in western China. The proposed model was tested using confirmatory factor analysis, hierarchical regression, and bootstrapping. The results indicate that work overload is positively associated with subjective well-being through job crafting, but negatively associated with subjective well-being through emotional exhaustion. Self-efficacy strengthens the positive association between work overload and job crafting while weakening the positive relationship between work overload and emotional exhaustion. Moreover, self-efficacy moderates both indirect associations, amplifying the beneficial pathway through job crafting and attenuating the detrimental pathway through emotional exhaustion. These findings reveal two distinct mechanisms linking work overload to rural teachers’ subjective well-being and extend research on occupational stress by identifying self-efficacy as an important boundary condition. The study also provides practical implications for educational authorities seeking to optimize workload arrangements and develop targeted support and incentive mechanisms for rural teachers.

## 1. Introduction

Work overload occurs when employees perceive that job demands exceed the time and resources available to meet them. It is commonly manifested in long working hours, excessive task demands, and substantial job responsibilities (Roberts et al., 1997). According to the 2018 OECD Teaching and Learning International Survey (TALIS), approximately 18% of teachers worldwide reported considerable work-related stress, with the proportion exceeding 30% in some countries (OECD, 2020). Such stress is closely associated with turnover intentions, as highly stressed teachers are nearly twice as likely to leave the profession as those reporting lower stress levels (OECD, 2020). Escalating occupational stress and teacher attrition have further aggravated the global teacher shortage, which UNESCO and other international organizations identify as a major obstacle to achieving inclusive and equitable quality education (UNESCO & International Task Force on Teachers for Education 2030, 2023). These challenges have become increasingly prominent amid rapid social change, rising public expectations, and increasingly complex teaching environments (Arbia et al., 2023). Against this background, sustaining teachers’ professional motivation and subjective well-being under demanding working conditions has become a critical issue in educational research (Ab. Wahab et al., 2024; Benevene et al., 2020). Teachers’ subjective well-being is closely associated not only with their personal and professional development but also with students’ academic achievement and the broader school environment, alongside differences in teaching effectiveness and work engagement (X. Wang et al., 2024). The problem is particularly salient in rural China. Rural teachers frequently face heavy teaching responsibilities, extensive non-teaching duties, complex student-management demands, and considerable pressure associated with school–family communication. The coexistence of these demands is often associated with persistent perceptions of work overload among rural teachers (Cheng et al., 2023). Nevertheless, teachers may exhibit different patterns of response under comparable workloads. For some teachers, heavier responsibilities may coexist with greater motivation and more proactive work behaviors, whereas for others, they may be associated with greater emotional exhaustion and burnout. These divergent responses highlight the importance of considering the dual nature of work overload. Parasuraman et al. (1996) defined work overload as the perception that job-role demands are excessive and cannot be completed within the available time. However, overload involves more than the objective quantity of work. Frankenhaeuser and Gardell (1976) distinguished quantitative workload, referring to the amount of work required within a given period, from qualitative workload, referring to task difficulty and complexity. They further proposed that higher workload is particularly likely to be associated with stress when employees perceive limited control over the pace and methods of their work. This argument is consistent with the cognitive appraisal theory of stress, which posits that individuals’ experiences associated with a stressor vary not only with its objective characteristics but also with their evaluations of its demands and their perceived coping capacity (Folkman et al., 1986). Accordingly, empirical evidence concerning the associations between work overload and employee outcomes remains mixed. A substantial body of research conceptualizes work overload as a resource-depleting stressor and has linked it to greater emotional exhaustion, lower work engagement, and poorer well-being (Karatepe, 2013; Matthews et al., 2014; Sandmeier et al., 2022). In contrast, other studies suggest that, under certain conditions, work overload may be appraised as a challenge stressor that may be associated with greater proactive coping and more favorable work-related outcomes (Ingusci et al., 2021). Thus, work overload may be associated with both detrimental and beneficial outcomes, depending on how employees interpret and respond to demanding work situations. Despite growing interest in employees’ responses to work overload, occupational health and stress research has predominantly emphasized its adverse effects, particularly those related to resource depletion (Brockbank & Feldon, 2024). Its potentially constructive associations have received comparatively limited attention (De Beer et al., 2016), and few studies have simultaneously considered the positive and negative pathways linking work overload to employee outcomes within a unified analytical framework. This limitation is particularly evident in research on rural teachers, whose demanding occupational context may be associated with both resource depletion and proactive adaptation. Accordingly, this study examines whether work overload is associated with rural teachers’ subjective well-being through distinct positive and negative pathways (Gilboa et al., 2008).

Cognitive appraisal theory of stress posits that individuals do not passively endure stressors but actively evaluate them through two stages. Primary appraisal concerns whether a stressor is perceived as a challenge or a threat, whereas secondary appraisal concerns whether sufficient resources are perceived to be available for coping. These appraisals are associated with subsequent coping strategies, including problem-focused and emotion-focused coping, as well as with different adaptive outcomes (Folkman et al., 1986). Applied to rural teachers, this perspective suggests that the associations between work overload and subjective well-being may vary according to how work overload is appraised. When work overload is perceived as a professional challenge, such an appraisal may be associated with greater use of proactive coping strategies, such as job crafting, which in turn is positively associated with subjective well-being (Tadić et al., 2015). By contrast, when workload is appraised as a hindrance, it is associated with greater emotional exhaustion (Webster et al., 2011), which is, in turn, associated with lower levels of subjective well-being (Hakanen & Schaufeli, 2012). Thus, work overload may exhibit a double-edged pattern of association with rural teachers’ subjective well-being through distinct adaptive and strain-related pathways (Ab. Wahab et al., 2024).

An important question concerns the boundary conditions under which work overload may exhibit a double-edged pattern of association with rural teachers’ subjective well-being. According to the cognitive appraisal theory of stress, secondary appraisal involves individuals’ evaluations of the coping resources available to manage a stressor and is closely related to how primary appraisal corresponds with subsequent coping responses (Folkman et al., 1986). When individuals perceive that they possess sufficient coping resources, they may be more likely to appraise stressors as challenges and to report constructive coping strategies. By contrast, lower levels of perceived coping resources may be associated with a greater likelihood of threat appraisal and more adverse emotional responses (Folkman et al., 1986; Seery, 2011). Self-efficacy, defined as an individual’s belief in their capacity to successfully perform a task, represents a key personal resource relevant to the secondary appraisal process (Bandura, 1977). It reflects a sense of capability and has been positively associated with proactive work behavior and performance (Roczniewska et al., 2020). Evidence from a survey of 580 university teachers in China showed that self-efficacy was significantly associated with greater innovative teaching behavior and stronger work motivation (Gai et al., 2026). Similarly, Skaalvik and Skaalvik (2007) found that teachers with high self-efficacy reported greater teaching enthusiasm and a stronger problem-solving orientation when confronted with work-related pressures. In contrast, lower self-efficacy was associated with a greater tendency to perceive such demands as threatening and with higher levels of emotional resource depletion. A study further identified teacher self-efficacy as the strongest negative correlate of emotional exhaustion and suggested that it may serve as a potential buffering resource in relation to exhaustion (Kuok et al., 2022). Taken together, these findings suggest that self-efficacy may represent an important boundary condition shaping the strength of the associations between work overload and rural teachers’ subjective well-being.

Drawing on the cognitive appraisal theory of stress, this study develops a moderated dual-mediation model and examines it among primary and secondary school teachers in rural western China. It addresses two questions: First, is work overload associated with rural teachers’ subjective well-being in a double-edged manner through parallel indirect associations involving job crafting and emotional exhaustion? Second, does self-efficacy moderate these indirect associations? By addressing these questions, this study advances a more nuanced understanding of the interrelationships among excessive job demands, teachers’ adaptive and strain-related responses, and subjective well-being. It also offers practical implications for designing teacher management systems that support personal coping resources and well-being.

## 2. Literature Review and Research Hypotheses

### 2.1. The Positive Association Between Work Overload and Rural Teachers’ Subjective Well-Being Through Job Crafting

Job crafting refers to individuals’ proactive modification of their work tasks, relationships, and perceptions to better align their jobs with their abilities, needs, and preferences (Tadić et al., 2015). Unlike traditional top-down job design, job crafting represents a bottom-up strategy that emphasizes individual initiative and autonomy in managing work demands. Rather than passively accepting workplace constraints, employees actively reshape the content and meaning of their work through self-initiated adjustments (Wrzesniewski & Dutton, 2001). For rural teachers confronted with substantial teaching and non-teaching responsibilities, job crafting may represent an important adaptive strategy associated with the management of occupational stress, professional development, and sustained career engagement.

Cognitive appraisal theory of stress suggests that individuals’ initial evaluation of stressors is closely related to their subsequent coping responses (Folkman et al., 1986). Work overload is characterized by excessive job demands under limited time and resource constraints and is often associated with greater perceived work pressure, as well as heightened requirements for efficiency, task management, and problem solving. As a potential work stressor, work overload may be perceived not only as a source of pressure and excessive demands but also, under certain conditions, as a challenge (Webster et al., 2011). Challenge appraisals of work overload may be associated with a greater tendency to adopt proactive, problem-focused coping strategies (Lazarus & Folkman, 1984). Specifically, individuals may adjust job tasks, optimize work processes, modify existing work methods, reorganize available resources, and seek additional support to cope with excessive demands; these self-initiated adjustments are consistent with job crafting behaviors (Petrou et al., 2012). Fritz and Sonnentag (2009) showed that daily work stressors were associated with proactive behaviors, including efforts to improve work methods and seek feedback. These findings suggest that stressors are not invariably associated with adverse outcomes; under certain conditions, they may also be associated with positive affect and proactive action (Parker & Collins, 2008). Similarly, survey evidence indicates that educators experiencing work overload reported a greater tendency to modify their job content and working methods when they perceived their work as meaningful (Wingerden & Poell, 2019). Such adjustments were associated with sustained professional engagement and greater resilience in dealing with career-related challenges (Bakker et al., 2012; Wingerden & Poell, 2019). Evidence from a survey of 7743 university teachers in China further showed that challenge stressors were positively associated with teaching engagement (Xu et al., 2023). Accordingly, among rural teachers, perceiving work overload as a challenge rather than solely as an excessive burden may be associated with a greater tendency to engage in job crafting, including adjusting teaching strategies, improving work processes, and seeking collaboration with colleagues. Such behaviors may involve redesigning classroom activities to accommodate students’ needs, streamlining homework assessment and feedback procedures, and sharing non-teaching responsibilities with colleagues (Zhang et al., 2025). Based on this reasoning, the following hypothesis is proposed:

**H1.**  *Work overload is positively associated with rural teachers’ job crafting.*

Teachers’ subjective well-being refers to a positive state of psychological functioning experienced by teachers within their work environment. It primarily comprises two core dimensions: teaching efficacy, defined as teachers’ perceived capability to successfully achieve instructional goals and effectively facilitate students’ learning; and school connectedness, referring to teachers’ sense of belonging and support within the school, as well as the positive relationships they experience with colleagues, students, and school administrators (Mankin et al., 2018; Renshaw et al., 2015). According to cognitive appraisal theory of stress, individuals’ evaluations of stressors are closely associated with their coping responses and adaptive outcomes (Lazarus & Folkman, 1984). When work overload is perceived as a challenge rather than a threat, such an appraisal may be associated with a greater tendency to adopt proactive, problem-focused coping strategies. Job crafting represents one such proactive strategy and involves self-initiated modifications to work tasks, interpersonal relationships, and perceptions of work. These adjustments may be associated with greater perceptions of control, competence, and meaningfulness, which are positively related to subjective well-being (Tims et al., 2013). Such associations may be particularly relevant for rural teachers, who often work in schools characterized by limited resources and insufficient organizational support. Under such conditions, proactive adjustments may be associated with more favorable perceptions of available resources and higher levels of psychological well-being (Zhang et al., 2025). Similarly, Wingerden and Poell (2019) found that teachers who reported greater adaptation of their work practices in response to professional challenges also exhibited higher levels of psychological resilience. Taken together, these findings suggest that job crafting is positively associated with resource-related psychological experiences and teachers’ subjective well-being. Accordingly, the following hypothesis is proposed:

**H2.**  *Job crafting is positively associated with rural teachers’ subjective well-being.*

Building on the preceding arguments, rural teachers who appraise work overload as a challenge may report a greater tendency to engage in job crafting. C. Zheng et al. (2024) identified four forms of job crafting: task crafting, relational crafting, cognitive crafting, and work–life crafting. In practice, teachers may redesign instructional activities and refine homework feedback practices, collaborate with colleagues to share non-teaching responsibilities, and reinterpret the meaning and value of their work. Such adjustments may be associated with a greater sense of control over work and more positive performance-related experiences, which, in turn, are positively related to professional identity and job satisfaction. Subjective well-being involves individuals’ positive cognitive evaluations of their lives, including their work experience (Diener, 1984). The greater sense of control and accomplishment associated with job crafting may also correspond with stronger satisfaction of the basic psychological needs for autonomy and competence (Deci & Ryan, 2000), both of which are positively related to positive affect and life satisfaction. Taken together, these arguments suggest that work overload may be indirectly and positively associated with rural teachers’ subjective well-being through job crafting. Based on this, the paper proposes the following hypotheses:

**H3.**  *Job crafting is expected to statistically mediate the positive association between work overload and rural teachers’ subjective well-being.*

### 2.2. The Negative Association Between Work Overload and Rural Teachers’ Subjective Well-Being Through Emotional Exhaustion

Emotional exhaustion refers to a state characterized by feelings of emotional and physical depletion. As a central component and an early manifestation of occupational burnout, it reflects perceived depletion of emotional resources in the context of prolonged work pressure (Maslach et al., 2001). Higher levels of emotional exhaustion are associated with poorer psychological functioning and lower levels of subjective well-being (Hakanen & Schaufeli, 2012). Emotionally exhausted teachers often report fatigue, diminished enthusiasm, and doubts about their professional competence. Such experiences of resource depletion are also associated with a reduced sense of meaning and accomplishment at work, weaker professional identity and commitment, and lower subjective well-being (Wei & Ye, 2022). Rural teachers may be particularly susceptible to emotional exhaustion because they are required to manage both routine teaching duties and numerous non-teaching responsibilities. The coexistence of these demands may be associated with sustained pressure on their emotional resources and greater perceived resource depletion (Ab. Wahab et al., 2024).

Cognitive appraisal theory of stress distinguishes between primary appraisal, through which individuals evaluate a stressor as a threat, harm, or challenge, and secondary appraisal, through which they assess their perceived coping capacity and available resources (Folkman et al., 1986). These appraisals are closely associated with subsequent emotional and behavioral responses. Threat appraisals are generally associated with greater reliance on emotion-focused coping strategies and with higher levels of negative emotional states, such as anxiety and helplessness (Fernandez De Henestrosa et al., 2023; Folkman et al., 1986; Hulin et al., 2024). Work overload is characterized by high job demands relative to the time, energy, and resources available to meet them and may therefore be associated with a perceived imbalance between work requirements and coping resources. Higher levels of work overload may also be associated with greater concerns about one’s capacity to cope effectively, as well as with higher levels of perceived resource depletion and exhaustion (De Beer et al., 2016; Sandmeier et al., 2022). Because work overload is often persistent, emotion-focused coping may be more closely related to the management of negative emotions than to changes in the underlying workload. Persistent work overload may therefore coexist with continued strain on emotional resources and higher levels of emotional exhaustion, a pattern consistent with the resource-loss perspective (Hobfoll, 1989). This pattern may be particularly relevant to rural teachers in China, whose responsibilities often extend beyond teaching to student safety management, administrative reporting, institutional inspections, document preparation, and other non-teaching duties. The coexistence of these demands may be associated with sustained emotional and physical resource investment and fewer opportunities for recovery. Prior research has likewise linked higher workload demands and extended working hours with greater emotional exhaustion among teachers (Magtalas, 2024; Sandmeier et al., 2022). Accordingly, the following hypothesis is proposed:

**H4.**  *Work overload is positively associated with emotional exhaustion among rural teachers.*

Emotional exhaustion is consistently associated with less favorable employee attitudes, behaviors, and well-being. Affective Events Theory suggests that workplace events, including excessive job demands, are associated with emotional responses that, in turn, relate to job satisfaction, work engagement, and turnover intentions (Weiss & Beal, 2005). As a persistent negative emotional state, emotional exhaustion may be associated with less favorable evaluations of work, including lower job satisfaction, weaker professional identity, reduced work enthusiasm and professional accomplishment, and stronger turnover intentions (Leiter & Maslach, 1988). Empirical evidence further supports a negative association between emotional exhaustion and well-being. M. Zhu et al. (2025) found that perceived time pressure was indirectly associated with lower subjective well-being through emotional exhaustion. Similarly, Skaalvik and Skaalvik (2007) reported a negative association between emotional exhaustion and teacher well-being among 244 primary and secondary school teachers in Norway, while a systematic review by Hascher et al. (2021) identified emotional exhaustion as a consistent negative correlate of teacher well-being. Taken together, these findings suggest that emotional exhaustion may represent an important pathway linking excessive work demands with lower teacher well-being. Therefore, the following hypothesis is proposed:

**H5.**  *Emotional exhaustion is negatively associated with rural teachers’ subjective well-being.*

Integrating these arguments, work overload may be indirectly and negatively associated with rural teachers’ subjective well-being through emotional exhaustion. When excessive work demands are appraised as threats, teachers may report greater anxiety and helplessness and a stronger tendency to rely on emotion-focused coping. Persistent work demands may also be associated with sustained emotional resource strain and higher levels of emotional exhaustion (Hobfoll, 1989; Maslach et al., 2001). In turn, emotional exhaustion is associated with lower levels of work meaning, accomplishment, and satisfaction, as well as weaker professional identity and lower subjective well-being (Leiter & Maslach, 1988; Skaalvik & Skaalvik, 2007; M. Zhu et al., 2025). Accordingly, the following mediation hypothesis is proposed:

**H6.**  *Emotional exhaustion statistically mediates the negative association between work overload and rural teachers’ subjective well-being.*

### 2.3. The Moderating Role of Self-Efficacy

Self-efficacy refers to individuals’ beliefs in their ability to accomplish specific tasks and mobilize the motivation, cognitive resources, and courses of action required to manage situational demands (M. Zhu et al., 2025). It is closely associated with behavioral choices, effort, persistence, and coping responses (Bandura, 1977). Within cognitive appraisal theory of stress, self-efficacy represents an important personal resource relevant to secondary appraisal, through which individuals evaluate whether they possess sufficient resources to manage a stressor (Lazarus & Folkman, 1984). Individuals with higher self-efficacy are more likely to perceive adequate coping resources and to appraise demanding situations as manageable challenges, whereas lower self-efficacy is associated with perceptions of insufficient resources and a greater tendency toward threat appraisal (Bandura, 1977; X. Wang et al., 2024). Self-efficacy may therefore condition the association between work overload and job crafting. Previous research has linked higher self-efficacy to greater engagement in job crafting, including increasing structural and social job resources, diversifying tasks, pursuing learning opportunities, and seeking challenging job demands (Miraglia et al., 2017; Rošková & Faragová, 2020; Tims et al., 2014). These findings suggest that self-efficacy not only promotes challenge appraisals of work overload but also provides the confidence needed to translate such appraisals into proactive job crafting. Among rural teachers, higher self-efficacy may similarly be associated with a greater tendency to respond to work overload through proactive adjustments to tasks, relationships, and work practices. By contrast, lower self-efficacy may be associated with less proactive adjustment when work demands are high. Thus, the positive association between work overload and job crafting may be stronger at higher levels of self-efficacy.

Self-efficacy may also condition the association between work overload and emotional exhaustion. Higher self-efficacy is generally associated with greater perceived coping capacity, whereas lower self-efficacy has been linked to stronger threat-focused cognitions and higher emotional exhaustion (Matthews et al., 2014; Skaalvik & Skaalvik, 2007; Zeng et al., 2024). For rural teachers experiencing work overload, higher self-efficacy may therefore correspond with lower emotional resource strain, whereas lower self-efficacy may be associated with greater vulnerability to emotional exhaustion. Accordingly, the positive association between work overload and emotional exhaustion may be weaker at higher levels of self-efficacy. Based on these arguments, the following hypotheses are proposed:

**H7.**  *Self-efficacy moderates the positive association between work overload and job crafting among rural teachers, such that this positive association is stronger at higher levels of self-efficacy.*

**H8.**  *Self-efficacy moderates the positive association between work overload and emotional exhaustion among rural teachers, such that this positive association is weaker at higher levels of self-efficacy.*

### 2.4. The Moderated Mediation Effect

Integrating H3 and H7, the positive indirect association between work overload and rural teachers’ subjective well-being through job crafting is expected to be stronger at higher levels of self-efficacy. Rural teachers with higher self-efficacy are more likely to perceive sufficient coping resources and to appraise work overload as a manageable challenge rather than a threat. Such appraisals are associated with greater engagement in job crafting, which, in turn, is positively associated with perceived control, successful coping experiences, and subjective well-being (Miraglia et al., 2017; Tims et al., 2014).

Integrating H6 and H8, the negative indirect association between work overload and subjective well-being through emotional exhaustion is expected to be weaker at higher levels of self-efficacy. Higher self-efficacy is associated with greater perceived coping capacity, lower threat perceptions, and less emotional resource strain under demanding work conditions (Cuadrado et al., 2024). Accordingly, the positive association between work overload and emotional exhaustion may be weaker among teachers with higher self-efficacy, resulting in a less negative indirect association between work overload and subjective well-being through emotional exhaustion. Based on these arguments, the following hypotheses are proposed:

**H9.**  *Self-efficacy moderates the positive indirect association between work overload and rural teachers’ subjective well-being through job crafting, such that this positive indirect association is stronger at higher levels of self-efficacy.*

**H10.**  *Self-efficacy moderates the negative indirect association between work overload and rural teachers’ subjective well-being through emotional exhaustion, such that this negative indirect association is weaker at higher levels of self-efficacy.*

**Figure 1:**
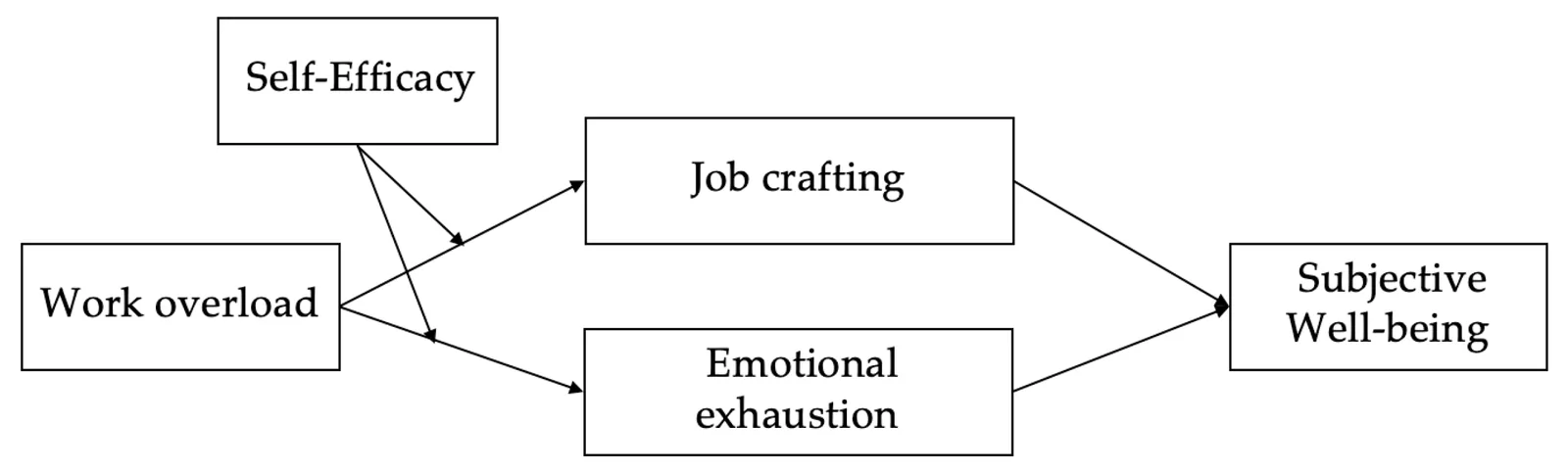
Theoretical model.

In summary, the proposed theoretical model is presented in Figure 1.

## 3. Methods

### 3.1. Participants and Data Collection

Data were collected through an online questionnaire from rural primary and secondary school teachers across 12 provincial-level administrative regions in western China, primarily via the Credamo platform (https://www.credamo.com/#/, accessed on 7 June 2026). Convenience and snowball sampling were used to broaden sample reach. Before participation, respondents were informed of the study objectives, confidentiality procedures, and relevant ethical considerations, and participation was voluntary. School affiliations were not systematically recorded; therefore, potential clustering of observations within schools could not be assessed or accounted for in the analyses. Rural teachers in western China were selected because they often encounter diverse and complex sources of work overload and experience relatively high levels of occupational stress, making them an appropriate population for examining the proposed relationships (Zeng et al., 2024; Zhao & Fu, 2018). Data collection was completed in June 2026. A total of 674 questionnaires were collected. Prior to data analysis, all responses were subjected to a data-quality screening procedure, resulting in the exclusion of 35 questionnaires. Specifically, 22 questionnaires were excluded because of excessively short completion times, defined as less than one-third of the sample median completion time, while 13 were excluded because of irregular response patterns, such as systematically alternating between response options or consistently selecting the midpoint option. After data screening, 639 valid responses were retained, yielding a valid response rate of 94.8%.

The regional distribution of the final sample was as follows: Gansu (*n* = 55, 8.6%), Guangxi (*n* = 63, 9.9%), Guizhou (*n* = 61, 9.5%), Inner Mongolia (*n* = 57, 8.9%), Ningxia (*n* = 48, 7.5%), Qinghai (*n* = 40, 6.3%), Shaanxi (*n* = 53, 8.3%), Sichuan (*n* = 55, 8.6%), Xizang (*n* = 44, 6.9%), Xinjiang (*n* = 49, 7.7%), Yunnan (*n* = 56, 8.8%), and Chongqing (*n* = 58, 9.1%). The demographic characteristics of the respondents are presented in Table 1.

### 3.2. Instruments

To enhance measurement reliability, all constructs were assessed using established scales from prior research. Because the survey was administered in China, two bilingual researchers independently translated and reviewed the scale items. Minor modifications were made to ensure that the items reflected the working context of rural teachers in China. Unless otherwise specified, all items were rated on a five-point Likert scale ranging from 1 (“strongly disagree”) to 5 (“strongly agree”).

#### 3.2.1. Work Overload

Work overload was measured using the eight-item scale developed by Cousins et al. (2004), which assesses teachers’ perceptions of excessive work demands. An overall work overload score was computed by averaging the item scores. A sample item is “My daily teaching responsibilities, including lesson preparation, classroom instruction, assignment grading, and student tutoring, often require me to work overtime.” The scale demonstrated good internal consistency in this study (Cronbach’s α = 0.925).

#### 3.2.2. Teachers’ Subjective Well-Being

The construct was assessed using an adapted version of the well-being questionnaire initially developed by Mankin et al. (2018), which has demonstrated satisfactory reliability among Chinese teachers (X. Wang et al., 2024). The adapted scale contains eight items. To align the instrument with the rural-school context, minor wording modifications were made without altering the substantive meaning of the items. For example, the original item “I feel like I belong at this school” was adapted to “I feel like I belong at this rural school”. Although the original scale comprises two related dimensions—teaching efficacy and school connectedness—the present study focuses on rural teachers’ overall subjective well-being rather than dimension-specific associations. Accordingly, the eight items were averaged to create a composite score representing overall subjective well-being. Because all items were rated on the same response scale, the mean score is a linear transformation of the summed score while retaining the original response metric, thereby facilitating interpretation. The scale demonstrated good internal consistency in the present sample (Cronbach’s α = 0.921).

#### 3.2.3. Job Crafting

Job crafting was measured using the six items representing the individual job-crafting dimension of the scale developed by Leana et al. (2009). Given that the present study conceptualizes job crafting as teachers’ self-initiated modifications to their own work tasks, relationships, and work practices, only the six items assessing individual job crafting were retained. The collaborative job-crafting dimension, which captures jointly coordinated changes undertaken with coworkers, was not included because it reflects a collective form of work redesign that falls outside the individual-level focus of the present study. Because the original scale was developed in a childcare context, minor wording adaptations were made to enhance its contextual relevance for rural teachers in western China while preserving the substantive meaning of the original items. Specifically, three items were slightly adapted. The original item, “Organize special events in your classroom (such as celebrating a child’s birthday, etc.) on your own.” was adapted to “I proactively organize special activities in my class, such as class meetings, student celebrations, or extracurricular learning activities.” The item, “On your own, bring in other materials from home for the classroom (such as empty jars or egg cartons)” was adapted to “I proactively seek out, bring in, or prepare additional teaching materials or locally available instructional resources for use in the classroom.” Finally, the original item, “Rearrange equipment or furniture in the play areas of your classroom on your own” was adapted to “I proactively rearrange the equipment, furniture, or learning spaces in my classroom to better support my teaching.” Following Leana et al. (2009), an overall job crafting score was calculated by averaging the scores across the six items, with higher scores indicating higher levels of job crafting among rural teachers. In the present study, the scale demonstrated good internal consistency (Cronbach’s α = 0.897).

#### 3.2.4. Emotional Exhaustion

Emotional exhaustion was measured using the five-item scale developed by Maslach and Jackson (1981), which assesses individuals’ feelings of fatigue and emotional depletion at work. An overall emotional exhaustion score was computed by averaging the item scores. A sample item is “I feel completely exhausted by my work.” The scale demonstrated good internal consistency in the present study (Cronbach’s α = 0.900).

#### 3.2.5. Self-Efficacy

Self-efficacy was assessed using an adapted version of the 10-item General Self-Efficacy Scale developed by Schwarzer and Jerusalem (1995). Minor modifications were made to ensure its relevance to the work context of rural teachers. The scale measures individuals’ confidence in their ability to manage tasks and overcome difficulties. An overall self-efficacy score was computed by averaging the item scores. A sample item is “If I make sufficient effort, I can solve most problems.” The scale demonstrated good internal consistency in this study (Cronbach’s α = 0.942).

#### 3.2.6. Control Variables

Given the occupational characteristics and work context of rural teachers, this study included age (30 years or younger = 1, 31–50 years = 2, and 51 years or older = 3), gender (male = 1, female = 0), marital status (married = 1, unmarried = 0), and educational attainment (junior college degree or below = 1, bachelor’s degree = 2, and master’s degree or above = 3) as control variables to account for their potential confounding effects on the relationships among the focal variables. The coding scheme for the above variables is consistent with established practices in prior research (Adomako et al., 2025; J. Zhu et al., 2024).

### 3.3. Data Analysis Strategy

Quantitative data were analyzed using IBM SPSS Statistics 27.0, AMOS 22.0, and the PROCESS macro 4.2. Descriptive statistics, reliability analyses, and hierarchical regression analyses were conducted in SPSS, whereas the measurement model was assessed using AMOS. Mediation effects were first examined using multiple linear regression analysis and were further assessed using PROCESS Model 4 with 5000 bootstrap resamples. Moderation effects were examined using multiple linear regression analysis and further illustrated through simple slope analyses. Prior to the moderation analyses, work overload and self-efficacy were mean-centered, and their interaction term was constructed using the mean-centered variables. Moderated mediation was subsequently examined using PROCESS Model 7 with 5000 bootstrap resamples.

## 4. Results

### 4.1. Reliability and Validity

Table 2 presents the reliability and convergent validity results for the study constructs. The Cronbach’s alpha coefficients for work overload, job crafting, emotional exhaustion, subjective well-being, and self-efficacy were 0.925, 0.897, 0.900, 0.921, and 0.942, respectively, all exceeding the recommended threshold of 0.70. In addition, the composite reliability (CR) values for all constructs were above 0.70, providing evidence of satisfactory internal consistency reliability. With respect to convergent validity, all standardized factor loadings exceeded 0.60, and the average variance extracted (AVE) for each construct was greater than 0.50. Taken together, these results indicate that the measurement scales demonstrated adequate reliability and convergent validity. Regarding discriminant validity, this study employed the Fornell–Larcker criterion and the heterotrait–monotrait (HTMT) ratio as assessment criteria, with the results presented in Table 2. The square root of the average variance extracted (AVE) for each construct exceeded its correlations with the other constructs, and all HTMT values were below the recommended threshold of 0.90. These results satisfied the commonly accepted criteria and provided evidence of adequate discriminant validity among the study constructs.

### 4.2. Common Method Bias Test

Harman’s single-factor test was conducted to assess potential common method bias. All items measuring work overload, job crafting, emotional exhaustion, subjective well-being, and self-efficacy were entered into an unrotated exploratory factor analysis. The first factor accounted for 30.751% of the total variance, below the commonly used threshold of 40% (Podsakoff et al., 2003), indicating that common method bias was unlikely to be a serious concern. As an additional robustness check, a common latent method factor was incorporated into the original measurement model. The changes in model fit were minimal (ΔRMSEA = 0.001, ΔCFI = 0.001, ΔTLI = 0.001, and ΔSRMR = 0.003). These results suggest that common method variance was unlikely to pose a substantial concern for the measurement model. Multicollinearity was also examined using variance inflation factors. All variance inflation factor (VIF) values were below 2 (maximum VIF = 1.99), indicating that multicollinearity was not a serious concern among the study variables.

### 4.3. Confirmatory Factor Analysis

Confirmatory factor analysis was conducted to assess the construct validity of the measurement model and the discriminant validity among work overload, job crafting, emotional exhaustion, self-efficacy, and subjective well-being. As shown in Table 3, the hypothesized five-factor model provided a better fit than the alternative models, with χ^2^/df = 1.296, CFI = 0.988, TLI = 0.987, RMSEA = 0.022, and SRMR = 0.028. These results indicated that the measurement model exhibited good overall fit and satisfactory discriminant validity.

### 4.4. Means, Standard Deviations, and Correlation Coefficients for Each Variable

Table 4 presents the descriptive statistics and correlations among the key variables. Work overload was positively correlated with job crafting (*r* = 0.274, *p* < 0.001) and emotional exhaustion (*r* = 0.254, *p* < 0.001). Job crafting was positively correlated with subjective well-being (*r* = 0.260, *p* < 0.001), whereas emotional exhaustion was negatively correlated with subjective well-being (*r* = −0.320, *p* < 0.001). These correlations were consistent with the hypothesized associations and provided preliminary support for the subsequent hypothesis tests.

### 4.5. Hypothesis Testing

#### 4.5.1. Testing of Main Effects and Mediating Effects

First, hierarchical regression analysis was conducted to examine the statistical mediating roles of job crafting and emotional exhaustion in the association between work overload and subjective well-being. The results are presented in Table 5. As shown in Model 1, work overload was positively associated with job crafting (*b* = 0.273, *p* < 0.001), supporting H1. When work overload and job crafting were simultaneously entered as predictors of subjective well-being in Model 6, job crafting was positively associated with subjective well-being (*b* = 0.341, *p* < 0.001), supporting H2. Moreover, the regression coefficient for work overload decreased from 0.252 (Model 5) to 0.159 (Model 6) after job crafting was included. This attenuation was consistent with a partial statistical mediating role of job crafting in the positive association between work overload and subjective well-being, providing preliminary support for H3.

As shown in Model 3, work overload was positively associated with emotional exhaustion (*b* = 0.271, *p* < 0.001), supporting H4. When work overload and emotional exhaustion were simultaneously entered as predictors of subjective well-being in Model 7, emotional exhaustion was negatively associated with subjective well-being (*b* = −0.372, *p* < 0.001), supporting H5. Moreover, after emotional exhaustion was included in the model, the coefficient for work overload increased from 0.252 (Model 5)to 0.353 (Model 7). This pattern was consistent with a negative indirect association between work overload and subjective well-being through emotional exhaustion. The change in the coefficient of work overload after emotional exhaustion was included in the model suggests that this mediating pattern may be inconsistent rather than a simple mediation pattern. The results therefore provided preliminary support for H6.

Second, the statistical mediating roles were further examined using a bootstrapping procedure. Specifically, 5000 bootstrap resamples were generated from the original sample, and the indirect association was estimated for each resample to construct a bootstrap sampling distribution and corresponding confidence interval. A statistically significant indirect association was inferred when the bias-corrected 95% confidence interval did not include zero. The results are presented in Table 6. The bootstrap estimate of the indirect association between work overload and subjective well-being through job crafting was 0.0560, with a 95% bootstrap confidence interval of [0.0323, 0.0827]. Because the confidence interval did not include zero, the results provided support for H3. Similarly, the bootstrap estimate of the indirect association through emotional exhaustion was −0.0789, with a 95% bootstrap confidence interval of [−0.1123, −0.0459]. This confidence interval also excluded zero, providing support for H6.

#### 4.5.2. Testing for Moderating Effects

We used hierarchical regression analysis to test the moderating effect of self-efficacy. As shown in Model 2 of Table 5, the interaction between work overload and self-efficacy was positive and statistically significant in relation to job crafting (*b* = 0.120, *p* < 0.001). This pattern was consistent with a stronger positive association between work overload and job crafting at higher levels of self-efficacy, thereby supporting H7.

**Figure 2:**
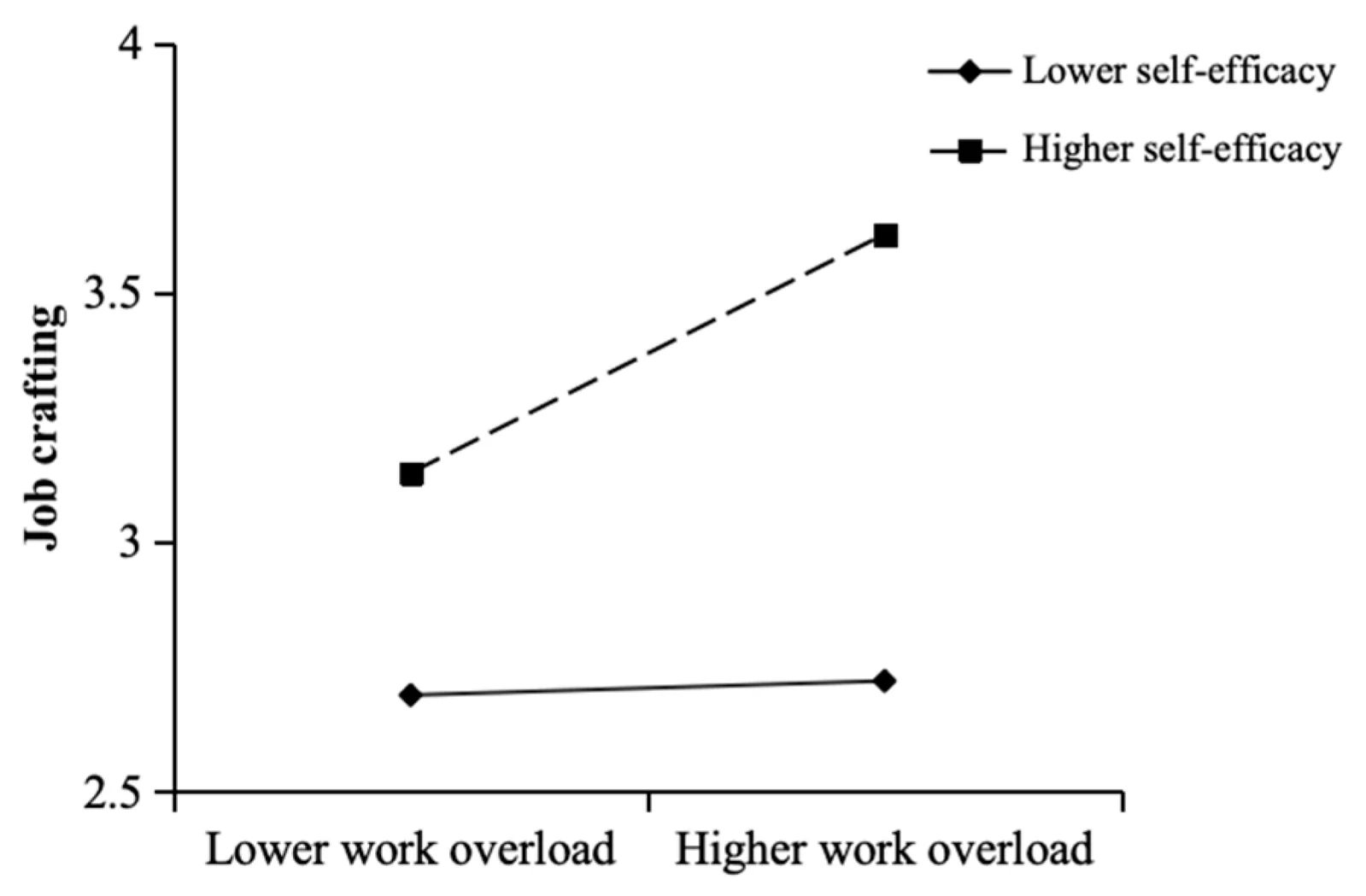
The moderating effect of self-efficacy between work overload and job crafting.

**Figure 3:**
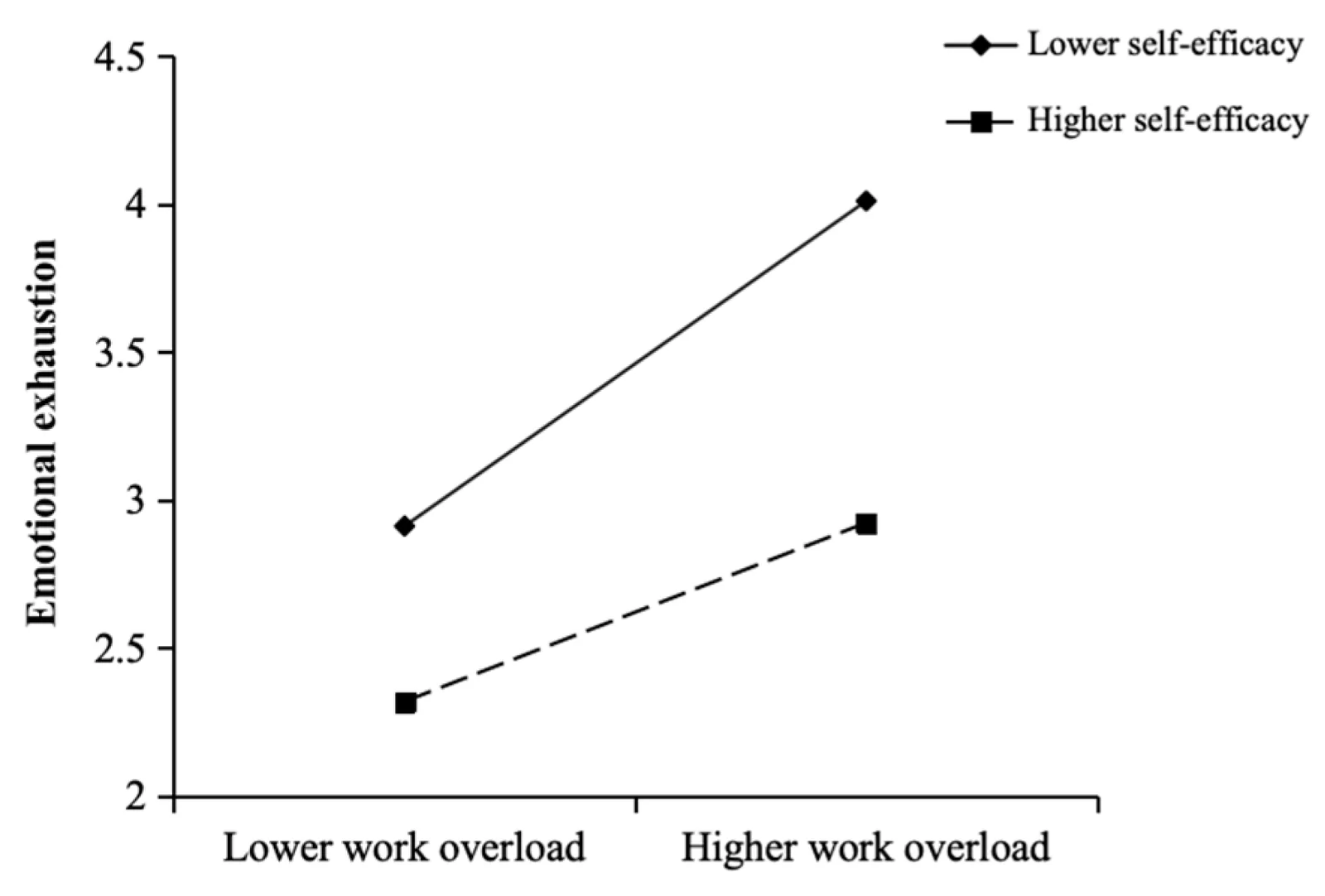
The moderating effect of self-efficacy between work overload and emotional exhaustion.

Model 4 further showed that the interaction term was negative and statistically significant in relation to emotional exhaustion (*b* = −0.132, *p* < 0.001), indicating that the positive association between work overload and emotional exhaustion was weaker at higher levels of self-efficacy. Thus, H8 was also supported. To further illustrate these moderating effects, simple slope analyses were conducted. As shown in Figure 2, the positive association between work overload and job crafting was stronger at higher levels of self-efficacy. Figure 3 showed that the positive association between work overload and emotional exhaustion was weaker at higher levels of self-efficacy. Together, these findings provided further support for H7 and H8.

#### 4.5.3. Testing of the Moderated Mediating Effect

Following the procedure suggested by Edwards and Lambert (2007), we first calculated the magnitude of indirect effects at “high(+SD)” and “low(−SD)” levels of moderating variables and then estimated the significance of these effects using the Bootstrapping approach. The results are presented in Table 6. For the pathway involving job crafting, the index of moderated mediation was 0.0246, with a 95% bootstrap confidence interval of [0.0088, 0.0446]. Because the confidence interval did not include zero, the results supported a statistically significant moderated mediation pattern. At low levels of self-efficacy, the conditional indirect association between work overload and subjective well-being through job crafting was not statistically significant, as the 95% bootstrap confidence interval included zero [−0.0210, 0.0259]. At high levels of self-efficacy, the conditional indirect association was positive and statistically significant (estimate = 0.0512, 95% bootstrap CI [0.0269, 0.0826]). The difference between the two conditional indirect effects was statistically significant (difference = 0.0480, 95% bootstrap CI [0.0172, 0.0871]). These findings were consistent with a stronger positive indirect association between work overload and subjective well-being through job crafting at higher levels of self-efficacy, thereby providing support for H9.

For the pathway involving emotional exhaustion, the index of moderated mediation was 0.0382, with a 95% bootstrap confidence interval of [0.0153, 0.0650]. Because the confidence interval did not include zero, the results supported a statistically significant moderated mediation pattern. At low levels of self-efficacy, the conditional indirect association between work overload and subjective well-being through emotional exhaustion was negative and statistically significant (estimate = −0.1662, 95% bootstrap CI [−0.2209, −0.1159]). At high levels of self-efficacy, this negative conditional indirect association was weaker (estimate = −0.0915, 95% bootstrap CI [−0.1304, −0.0552]). The difference between the two conditional indirect effects was statistically significant (difference = 0.0747, 95% bootstrap CI [0.0298, 0.1269]). These findings were consistent with a weaker negative indirect association between work overload and subjective well-being through emotional exhaustion at higher levels of self-efficacy, thereby providing support for H10.

## 5. Discussion

In an increasingly demanding educational environment, rural teachers are often required to manage overlapping instructional and non-instructional responsibilities and may consequently experience persistently high workloads. However, their psychological and behavioral responses to work overload vary considerably. Existing research has generally examined the positive and negative outcomes associated with work overload separately, with limited attention to their coexistence within a unified theoretical framework. Moreover, the few studies that have considered both pathways have focused primarily on corporate employees (De Beer et al., 2016; Gilboa et al., 2008), leaving rural teachers comparatively underexplored. By simultaneously examining the potentially beneficial and detrimental pathways linking work overload with subjective well-being, this study extends the literature on teacher well-being and provides a more nuanced understanding of the different ways in which rural teachers may respond to excessive work demands.

First, this study highlights the dual associations between work overload and rural teachers’ subjective well-being, suggesting that excessive work demands may be linked to both potentially beneficial and detrimental outcomes. The organizational management literature offers two competing perspectives on work overload. One perspective conceptualizes work overload as a challenge stressor that may be associated with proactive behavior and creativity (Fritz & Sonnentag, 2009), whereas the other adopts a more threat-oriented view and emphasizes its associations with adverse employee outcomes, including poorer physical and psychological health, burnout, and turnover intentions (De Beer et al., 2016; Karatepe, 2013; Matthews et al., 2014). However, these two perspectives have rarely been considered together within a unified framework. In the field of education, although some studies have begun to examine the psychological and behavioral correlates of teachers’ work overload, most have focused primarily on its negative associations with teacher well-being, including higher stress, poorer physical and mental health, greater burnout, and heightened uncertainty (Arbia et al., 2023). Comparatively little attention has been paid to the possibility that work overload may also be associated with potentially beneficial aspects of teachers’ subjective well-being (Ortan et al., 2021). Given that subjective well-being has been regarded as an important link between teachers’ work-related functioning and educational quality, it is of substantial relevance to both individuals and educational organizations. Ab. Wahab et al. (2024) therefore called for greater attention to both the potentially positive and negative associations between work overload and teacher well-being. Responding to this gap, the present study integrates potentially beneficial and detrimental pathways within a single framework, providing a more comprehensive account of the positive and negative associations between work overload and rural teachers’ subjective well-being. These findings not only broaden understanding of the factors associated with teachers’ subjective well-being but also extend research on outcomes associated with work overload beyond conventional organizational settings.

Second, this study identifies two contrasting pathways linking work overload to rural teachers’ subjective well-being, providing an integrated account of the ambivalent psychological responses and dual mechanisms that may arise when employees are confronted with excessive work demands. One pathway is potentially beneficial and operates through job crafting, whereas the other is detrimental and operates through emotional exhaustion. The results showed that work overload was positively associated with job crafting, which, in turn, was positively associated with subjective well-being. This pattern is consistent with a challenge-oriented interpretation in cognitive appraisal theory of stress. To date, only a limited number of studies have examined the potentially positive effects of work overload from a challenge-oriented perspective (Karatepe et al., 2014). According to this theory, when individuals appraise workplace stressors as challenge stressors, they tend to focus on the problem itself and actively cope with stressful events by modifying their own behaviors (Lazarus & Folkman, 1984). Under such circumstances, job crafting, as a proactive form of work-related behavior, is more likely to emerge. As a proactive coping strategy (Parker & Collins, 2008), job crafting may involve adjusting work methods, improving the use of teaching resources, streamlining work processes, and seeking collegial support, which may be associated with greater perceived control, confidence, and well-being (X. Wang et al., 2024; Wingerden & Poell, 2019). X. Zheng et al. (2024) likewise reported a positive association between job crafting and teachers’ psychological well-being. The findings of the present study are broadly consistent with the main conclusions reported in previous research. Importantly, the positive association between work overload and job crafting does not imply that excessive workload automatically elicits proactive job crafting. Importantly, however, the positive association between work overload and job crafting should not be interpreted as suggesting that excessive workload automatically elicits proactive job crafting. Job crafting requires employees to have sufficient discretion and resources to modify aspects of their work. Petrou et al. (2012) found that high daily work pressure was associated with greater resource seeking particularly when job autonomy was also high, while Berg et al. (2010) showed that opportunities for job crafting are shaped by employees’ structural positions and prescribed job boundaries. These findings suggest that job crafting under high workload depends on sufficient personal and job resources. This is particularly relevant in rural schools, where limited staffing, time, and material resources may constrain teachers’ capacity to redesign their work. In a teacher sample, Bakker et al. (2007) found that supervisor support, appreciation, innovativeness, and organizational climate were especially valuable under high job demands. Accordingly, the positive association observed here may be more likely when teachers retain sufficient autonomy, support, and confidence to cope with demanding tasks. Therefore, these findings also highlight the need to identify the boundary conditions that may shape the aforementioned pathways.

By contrast, work overload was positively associated with emotional exhaustion, which was negatively associated with subjective well-being, a pattern consistent with a threat-oriented interpretation. According to cognitive appraisal theory of stress, when individuals perceive a workplace stressor as potentially detrimental to their well-being and believe that they lack sufficient resources or capabilities to cope with it, they are likely to appraise the stressor as a threat. Such a threat appraisal may direct greater attention toward the potential losses and harm associated with the stressor (Lazarus & Folkman, 1984), thereby contributing to emotional exhaustion. Prior research has similarly linked excessive job demands to emotional exhaustion (Aboagye et al., 2024; Sandmeier et al., 2022) and emotional exhaustion to lower teacher well-being (Skaalvik & Skaalvik, 2018; M. Zhu et al., 2025). Compared with previous research, a notable contribution of the present study lies in explicitly incorporating emotional exhaustion into the association between work overload and teachers’ subjective well-being, thereby providing a more integrated and sequential account of the relationships among these three constructs. Because challenge and threat appraisals were not directly measured, however, these findings should be viewed as consistent with cognitive appraisal theory rather than as direct evidence that rural teachers appraised work overload as a challenge or threat. Future research should directly assess challenge and threat appraisals to examine whether they help account for these associations.

Third, this study identifies self-efficacy as an important boundary condition in the associations linking work overload to rural teachers’ subjective well-being. Specifically, self-efficacy moderated both indirect pathways, extending existing research on the role of individual psychological resources. Previous research has called for greater attention to factors that may mitigate the adverse effects of work stress (Bakker et al., 2007). However, prior studies on how teachers cope with work overload have primarily focused on contextual factors, such as job autonomy and perceived organizational support (Abdulaziz et al., 2022). By contrast, the potential moderating role of teacher self-efficacy has received comparatively limited empirical attention, and systematic evidence regarding its moderating effects remains scarce. The results showed that the positive indirect association between work overload and subjective well-being through job crafting was stronger at higher levels of self-efficacy. This pattern is consistent with the view that teachers with greater self-efficacy may be better positioned to respond proactively to demanding work situations (Keller-Schneider, 2024). Their greater confidence in managing work demands may be associated with more active job crafting, including adjustments to tasks, work methods, and interpersonal relationships, which in turn is associated with greater perceived accomplishment, control, and subjective well-being. By contrast, the negative indirect association through emotional exhaustion was weaker at higher levels of self-efficacy. Teachers with greater self-efficacy may have stronger confidence in managing demanding situations and using effective coping strategies, which may be associated with lower levels of emotional exhaustion (Cuadrado et al., 2024; Keller-Schneider, 2024). Conversely, lower self-efficacy may coincide with greater vulnerability to emotional resource depletion under heavy workloads (Cuadrado et al., 2024; Dung et al., 2024; Pei et al., 2024). Taken together, these findings suggest that self-efficacy may serve as an important psychological resource shaping how rural teachers respond to work overload. This finding further enriches the theoretical understanding of the relationship between the work environment and teacher well-being and provides a theoretical basis for school management practices aimed at mitigating the adverse effects of work overload by strengthening teachers’ self-efficacy.

## 6. Conclusions

### 6.1. Main Research Conclusions

Drawing on the cognitive appraisal theory of stress, this study developed a double-edged-sword model using data from 639 rural teachers across 12 provincial-level regions in western China. The results showed a positive indirect association between work overload and subjective well-being through job crafting, alongside a negative indirect association through emotional exhaustion. Moreover, the positive indirect association through job crafting was stronger at higher levels of self-efficacy, whereas the negative indirect association through emotional exhaustion was weaker at higher levels of self-efficacy, indicating statistically significant moderated mediation patterns.

### 6.2. Practical Implications

This study also offers several practical implications. First, given the double-edged associations between work overload and rural teachers’ subjective well-being, governments, educational authorities, and schools should approach workload-related policies with caution. Work arrangements should be introduced gradually and aligned with local educational conditions and teachers’ professional development needs (Y. Wang et al., 2025). Second, greater attention should be given to supporting rural teachers’ self-efficacy. Teachers may benefit from reflecting on effective work strategies, identifying constructive approaches to managing pressure, and building confidence through successful coping experiences. Access to external resources, including professional training, open educational materials, and support from schools and colleagues, may also be valuable for strengthening perceived coping resources and supporting teachers’ occupational well-being. Third, schools should provide job-crafting and emotional support tailored to rural teachers’ needs. Targeted guidance may support teachers in adjusting tasks, improving work processes, and developing appropriate coping strategies rather than relying on uniform interventions. Peer-support and mentoring arrangements may also provide opportunities for experience sharing and help address the emotional strain associated with professional isolation. More broadly, schools should foster a supportive culture that encourages constructive experimentation, tolerates reasonable mistakes, and adopts a developmental rather than purely accountability-oriented approach to teacher evaluation. Such organizational conditions may provide a more supportive context for teachers’ subjective well-being and professional development.

### 6.3. Limitations and Future Directions

Despite its contributions, this study has several limitations. First, although it focused on individual cognitive appraisal and coping mechanisms, school-level factors—such as principal leadership, organizational support, and collegial collaboration—may also shape the relationship between work overload and rural teachers’ subjective well-being. For example, principal instructional leadership may promote teachers’ job crafting by enhancing their psychological well-being. Future research could therefore incorporate multilevel factors to provide a more comprehensive explanation. Second, another limitation is that participants’ school affiliations were not systematically recorded, preventing us from assessing potential clustering of observations within schools. If responses were correlated within schools, the estimated standard errors may have been underestimated, potentially affecting the precision of the statistical inferences. Future research should record school-level identifiers and account for such clustering using appropriate analytical approaches. Third, all participants were rural teachers from western China recruited through convenience and snowball sampling, which may limit sample representativeness and the generalizability of the findings, particularly because teachers from more remote, smaller, or resource-constrained schools may be underrepresented. In addition, all variables were collected through cross-sectional self-report questionnaires, which may be subject to social desirability bias and do not permit causal inferences regarding the relationships among the study variables. Accordingly, caution is warranted when interpreting these associations and generalizing the findings to other regions or educational contexts. Future research could employ more rigorous sampling strategies, longitudinal or experimental designs, and multi-source or objective measures to further assess the robustness and external validity of the findings.

## Figures and Tables

**Table 1:** Sample characteristics.

Variable	Category	Frequency	Percentage (%)
Gender	Male	307	48.04%
	Female	332	51.96%
Age	30 years or younger	167	26.13%
	31–50 years old	240	37.56%
	51 years or older	232	36.31%
Marital status	Married	473	74.02%
	Unmarried	166	25.98%
Educational level	Junior college degree or below	210	32.86%
	Bachelor’s degree	400	62.60%
	Master’s degree or above	29	4.54%

**Table 2:** Reliability and validity.

Construct	Factor Loading	Cronbach’s α	AVE	CR	1	2	3	4	5
1. Work overload	0.771–0.790	0.925	0.610	0.925	**0.781**	0.301	0.278	0.282	0.351
2. Job crafting	0.735–0.782	0.897	0.592	0.897		**0.769**	0.372	0.436	0.451
3. Emotional exhaustion	0.798–0.812	0.900	0.643	0.900			**0.802**	0.351	0.319
4. Subjective well-being	0.747–0.791	0.921	0.594	0.921				**0.771**	0.455
5. Self-efficacy	0.767–0.805	0.942	0.620	0.942					**0.787**

Note: AVE, average variance extracted; CR, composite reliability; The diagonal entries represent the square roots of the average variance extracted (AVE), as specified by the Fornell–Larcker criterion, whereas the values above the diagonal represent the heterotrait–monotrait (HTMT) ratios.

**Table 3:** Confirmatory factor analysis.

Model		χ^2^/df	CFI	TLI	RMSEA	SRMR
Five Factors	WO; SWB; JC; EE; SE	1.296	0.988	0.987	0.022	0.028
Four Factors	WO; SWB + JC; EE; SE	3.993	0.876	0.868	0.068	0.089
Three Factors	WO; SWB + JC + EE; SE	6.486	0.772	0.758	0.093	0.113
Two Factors	WO; SWB + JC + EE + SE	10.005	0.625	0.602	0.119	0.137
Single Factor	WO + SWB + JC + EE + SE	14.278	0.446	0.414	0.144	0.169

Note: WO = Work Overload; SWB = Subjective Well-Being; JC = Job Crafting; EE = Emotional Exhaustion; SE = Self-Efficacy.

**Table 4:** Descriptive statistics and correlation analysis.

Variables	1	2	3	4	5	6	7	8	9
1. Gender									
2. Age	−0.013								
3. Educational level	−0.046	−0.223 ***							
4. Marital status	−0.009	0.692 ***	−0.132 ***						
5. Work overload	−0.026	0.031	0.013	0.036					
6. Job crafting	0.031	0.001	0.081	0.001	0.274 ***				
7. Emotional exhaustion	−0.077	0.003	−0.061	0.033	0.254 ***	−0.335 ***			
8. Subjective well-being	0.021	0.024	0.042	0.034	0.260 ***	0.397 ***	−0.320 ***		
9. Self-efficacy	0.078 *	0.002	0.039	−0.008	0.327 ***	0.415 ***	−0.294 ***	0.424 ***	
Mean	0.48	2.10	1.72	0.74	3.352	3.321	2.708	3.286	3.304
SD	0.500	0.784	0.542	0.439	0.961	0.956	1.033	0.932	0.976

Note: * *p* < 0.05, *** *p* < 0.001; The values below the diagonal represent the correlation coefficients among the variables.

**Table 5:** Results of hierarchical regressions.

Variables	Job Crafting		Emotional Exhaustion		Subjective Well-Being			
	Model 1	Model 2	Model 3	Model 4	Model 5	Model 6	Model 7	Model 8
Intercept	2.091 ***	3.043 ***	2.184 ***	3.041 ***	2.221 ***	1.507 ***	3.034 ***	2.426 ***
Gender	0.081	0.025	−0.152	−0.082	0.057	0.029	0.000	−0.004
Age	0.022	0.010	−0.080	−0.065	0.011	0.003	−0.019	−0.017
Marital status	−0.023	0.008	0.131	0.094	0.052	0.060	0.101	0.095
Educational Level	0.144	0.118	−0.141	−0.109	0.078	0.028	0.025	0.007
Work overload	0.273 ***	0.132 ***	0.271 ***	0.443 ***	0.252 ***	0.159 ***	0.353 ***	0.275 ***
Self-efficacy		0.344 ***		−0.431 ***				
Work overload × Self-efficacy		0.120 ***		−0.132 ***				
Job crafting						0.341 ***		0.206 ***
Emotional exhaustion							−0.372 ***	−0.291 ***
R-square	0.083	0.212	0.076	0.243	0.071	0.183	0.228	0.262
Adjusted R-square	0.076	0.203	0.069	0.235	0.064	0.176	0.221	0.253
F	11.465	24.189	10.330	28.972	7.890	16.943	21.098	20.714

Note: *** *p* < 0.001; All coefficients reported in the table are unstandardized coefficients.

**Table 6:** Results of mediating effect and moderated mediating effect.

Path	Estimate	S.E.	95% CI	
			LLCI	ULCI
Mediating effect				
WO→JC→SWB	0.0560	0.0129	0.0323	0.0827
WO→EE→SWB	−0.0789	0.0159	−0.1123	−0.0459
Moderated Mediating Effect				
WO→JC→SWB				
Lower(M − 1SD)	0.0032	0.0118	−0.0210	0.0259
Higher(M +1SD)	0.0512	0.0143	0.0269	0.0826
Differences	0.0480	0.0179	0.0172	0.0871
Index of moderated mediation	0.0246	0.0092	0.0088	0.0446
WO→EE→SWB				
Lower(M − 1SD)	−0.1662	0.0271	−0.2209	−0.1159
Higher(M +1SD)	−0.0915	0.0190	−0.1304	−0.0552
Differences	0.0747	0.0247	0.0298	0.1269
Index of moderated mediation	0.0382	0.0127	0.0153	0.0650

Note: CI, confidence interval; bootstrap confidence intervals are percentile-based; WO = Work Overload; SWB = Subjective Well-Being; JC = Job Crafting; EE = Emotional Exhaustion; S.E. = Self-Efficacy.

## Data Availability

The data presented in this study are available on request from the corresponding author due to confidentiality and privacy reasons.
